# Structural and functional attributes of malaria parasite diadenosine tetraphosphate hydrolase

**DOI:** 10.1038/srep19981

**Published:** 2016-02-01

**Authors:** Arvind Sharma, Manickam Yogavel, Amit Sharma

**Affiliations:** 1 Structural and Computational Biology Group, International Centre for Genetic Engineering and Biotechnology (ICGEB), Aruna Asaf Ali Road, New Delhi, 110067, India

## Abstract

Malaria symptoms are driven by periodic multiplication cycles of *Plasmodium* parasites in human red blood corpuscles (RBCs). Malaria infection still accounts for ~600,000 annual deaths, and hence discovery of both new drug targets and drugs remains vital. In the present study, we have investigated the malaria parasite enzyme diadenosine tetraphosphate (Ap4A) hydrolase that regulates levels of signalling molecules like Ap4A by hydrolyzing them to ATP and AMP. We have tracked the spatial distribution of parasitic Ap4A hydrolase in infected RBCs, and reveal its unusual localization on the infected RBC membrane in subpopulation of infected cells. Interestingly, enzyme activity assays reveal an interaction between Ap4A hydrolase and the parasite growth inhibitor suramin. We also present a high resolution crystal structure of Ap4A hydrolase in apo- and sulphate- bound state, where the sulphate resides in the enzyme active site by mimicking the phosphate of substrates like Ap4A. The unexpected infected erythrocyte localization of the parasitic Ap4A hydrolase hints at a possible role of this enzyme in purinerigic signaling. In addition, atomic structure of Ap4A hydrolase provides insights for selective drug targeting.

Malaria causes >200 million infections and ~600,000 deaths annually^1^. This infection is caused by one of five members of *Plasmodium* in humans, where *P. falciparum* (*Pf*) causes the most severe form of malaria. Escalating resistance in parasite against known drugs in clinical use necessitates discovery of novel drug targets that can be used in future^1^. *P. falciparum* life cycle shuttles between the female *Anopheles* mosquito and human host by a series of complex progressions within varying cellular milieus of hepatocytes, erythrocytes and mosquito gut etc.^2^,^3^. These developments require a tight coordination of parasite’s intracellular processes with changing environments that are primarily orchestrated by the multiple signalling pathways within malaria parasites^3^. Diadenosine tetraphosphate (Ap4A) is a ubiquitous signalling molecule present among eukaryotes, bacteria, archaea and viruses, and is well documented to participate in both intra- and extracellular signalling^4^,^5^,^6^. This molecule is a member of naturally occurring group of compounds, the dinucleoside 5′, 5^*n*^′-*p*^1^, *p*^*n*^-polyphosphates; Np_*n*_N’s (where N and N′ are 5′-*O*-nucleosides and *n* is the number of phosphate residues in the polyphosphate chain linking two 5′-esterified nucleosides). Diadenosine polyphosphates (Ap_*n*_A’s, n = 3–6) are predominantly synthesized as protein synthesis by-products by some of the members of aminoacyl-tRNA synthetase family (aaRSs), where Ap3A and Ap4A are the most prominent cellular forms^4^,^5^,^6^. Production of Ap4A by aaRSs is elevated during stress conditions and in mammals by a phosphorylation-induced nuclear migration of lysine-tRNA synthetase (KRS)^7^,^8^,^9^.

The Np_*n*_N, Ap4A, Ap5A and Gp4G levels within cells are primarily maintained by Nudix hydrolase superfamily member Ap4A hydrolase (Ap4AH from hereon). The Mg^2+^ -dependent Nudix hydrolase superfamily is recognized by a signature 23 amino acid Nudix motif G-x(5)-E-x(5)-[UA]-x-R-E-x(2)-E-E-x-G-U where U is an aliphatic, hydrophobic residue^8^,^9^,^10^. The consensus Nudix structural motif is located on a loop-helix and the signature Nudix fold has an α-β-α sandwich architecture^8^,^10^. Ap4AHs are phylogenetically classified into two distinct groups, animal-archeal type and the plant-bacterial type enzymes. Based on sequence analysis the *P. falciparum* Ap4AH (*Pf*Ap4AH, *EC 3.6.1.17*) was predicted to be an animal-archeal type which cleaves polyphosphate chain at the fourth phosphate from the tightly bound adenosine resulting in asymmetrical cleavage of Ap4A. This is distinct from some plant-bacterial type hydrolases^8^,^9^,^11^. Eukaryotic Ap4AHs are predominantly cytoplasmic or nuclear, while the bacterial Ap4AHs appear to be ribosome associated^12^,^13^,^14^. Levels of Ap4A in a cell are largely regulated by the synthesis and hydrolysis dynamics of KRS (which synthesises ~80% cellular Ap4A) and Ap4A hydrolase^7^,^8^,^15^. Intracellular Ap4A levels can influence physiological processes such as DNA repair, DNA replication, apoptosis and ion channel regulation^5^. In contrast, the extracellular Ap4A act as a cytokine-like molecule and via purinergic signalling pathway it participate in modulation of various physiological processes such as neurotransmission, cardiac electrophysiology, vasodilation and cellular communications, most notably between blood cells^5^,^6^. Cumulative studies of pathogenic bacteria and viruses have suggested possible role for Ap4AH in invasion of human cells^8^,^16^,^17^,^18^. Ap4AH associations with epithelial cell or erythrocyte invasion processes of *Bartonella bacilliformis*, *Escherichia coli* K1, *Actinobacillus actinomycetemcomitans, Salmonella enterica* and *Rickettsia prowazekii* are of particular interest for the present work^8^,^17^,^18^.

The aaRSs are main source of Ap_*n*_A’s and have been comprehensively characterized in malaria parasite for their biology and potential as drug targets^19^,^20^,^21^,^22^,^23^,^24^,^25^,^26^. These studies have shown that a reduced array of aaRSs are present in *P. falciparum*, where individual members have evolved to meet parasite specific needs, a feature that we also noticed for Nudix hydrolases in the present study^19^,^20^,^21^,^22^,^23^,^24^,^25^,^26^. It was recently shown that Ap4A can be produced in *P. falciparum* by lysine-tRNA synthetase (*Pf*KRS)^23^. Also, the enzyme kinetics and reaction specificities of *Pf*Ap4AH have been studied previously^11^. *Pf*Ap4AH can asymmetrically hydrolase Ap4A and Ap5A molecules produced by *Pf*KRS (and probably by other *Pf* aaRSs) to ATP + AMP and ATP + ADP, respectively^8^,^10^. In our analysis, we found that *P. falciparum* possesses a diminished set of these enzymes which is distinct from a related apicomplexan parasite *Toxoplasma gondii* (*Tg*). We also show atypical expression and localization profile of *Pf*Ap4AH, which indicates post-translational modification of native enzyme and a cytoplasmic localization in blood stage parasites along with unusual presence on RBC membrane during the feeding (trophozoite) and multiplication stages (schizont) of parasite. We further show that suramin can weakly inhibit *Pf*Ap4AH at an IC_50_ value of approximately ~11.8 μM and that it binds *Pf*Ap4AH with dissociation constant (K_d_) value of ~18 μM. We also provide two crystal structures of *Pf*Ap4AH - in apo and sulphate-bound forms at atomic resolution. Finally, we provide a comprehensive comparison between human and parasite Ap4AHs and discuss key active site differences which can be used for structure based drug design.

## Results

### *P. falciparum* possesses a diminished set of Nudix hydrolases

Genes encoding Nudix hydrolases in two apicomplexan parasites *P. falciparum* and *Toxoplasma gondii* were searched and identified as described in methods section. Nudix hydrolases vary in number from 0 to 30 in organisms (human- 24, *E. coli*- 12), where parasitic organisms have been documented to possess either very less or no members of this family^8^. Our analysis shows that *P. falciparum* and *T. gondii* contain reduced and distinct sets of five Nudix hydrolases in their genome (Table 1). The localization predictions suggest different spatial distribution schemes for *Tg* and *Pf*Ap4AH, where *T. gondii* enzyme maybe dually located in mitochondria and apicoplast while the *P. falciparum* enzyme is nuclear (Table 1). The observed disparity in evolutionary terms indicates selective retention and deletion of Nudix hydrolases post evolutionary branching of apicomplexan members *P. falciparum* and *T. gondii*. Other Ap4A hydrolases such ectonucleotide pyrophosphatase/phosphodiesterase family members were not found in *P. falciparum* suggesting that *Pf*Ap4AH could be the only enzyme responsible for Ap4A hydrolysis in parasite cell.

### *Pf*Ap4AH has unusual native expression and localization

**Figure 1:**
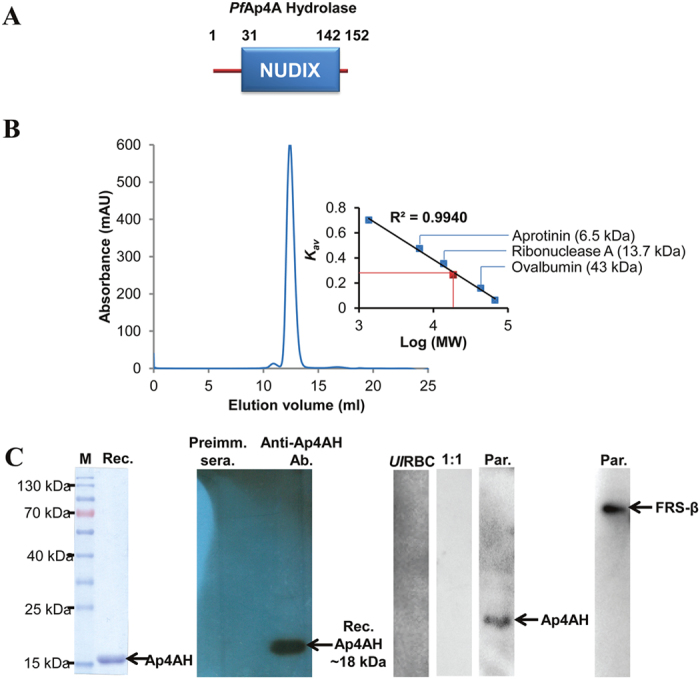
Domain Structure and expression of *Pf*Ap4AH. (**A**) Domain diagram of *Pf*Ap4AH. (**B**) Gel permeation chromatography profile of *Pf*Ap4A using Superdex 75 10/300 GL column (GE). Protein elutes at a volume corresponding to molecular weight of a monomer. Comparison with standard markers show that protein (shown as red dot on calibration curve) is a monomer. (**C**) Purified recombinant protein, and expression in parasite lysate using western blot are shown. Recombinant protein and molecular weight marker are denoted by Rec and M respectively. Recombinant protein was probed in westerns using preimmune sera (Preimm. Sera) purified specific antibodies (Anti-*Pf*Ap4AH ab). Antibodies were also used to probe protein in uninfected RBCs (*UI*RBCs) and parasite lysate (Par). 1:1 indicates competitive western where antibodies were pre-incubated with pure protein in 1:1 molar ratio prior to experiment. Phenylalanine-tRNA synthetase beta subunit (FRS-β) is used as a loading control.

**Figure 2:**
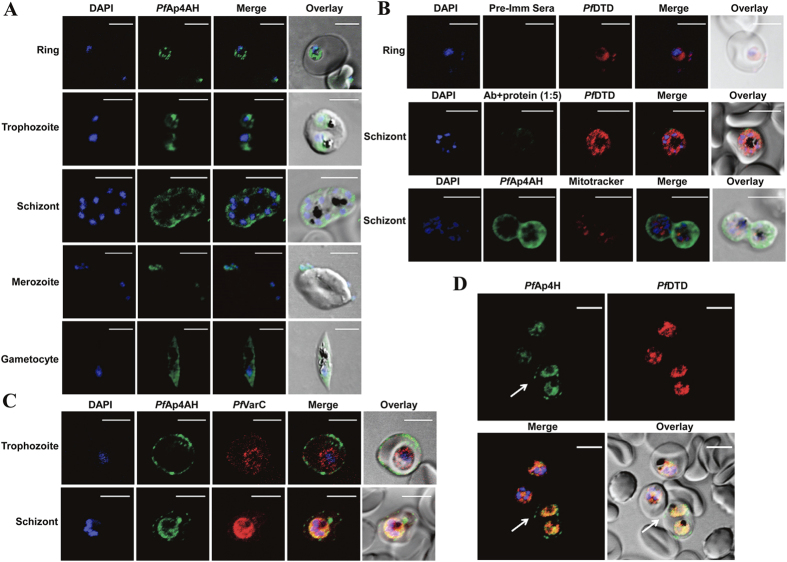
Spatial distribution of *P. falciparum* Ap4AH during erythrocytic schizogony. Shown are DAPI staining of nucleus in blue and *Pf*Ap4AH stained with Alexa 488 in green. (**A**) Confocal microscopy-data based spatial distribution of *Pf*Ap4AH in infected RBCs. *Pf*Ap4AH is non-nuclear in blood stages of the parasite and resides in its cytoplasm. (**B**) Non-mitochondrial localization with various controls is shown. Upper panel shows pre-immune serum (Pre-Imm Sera) control which does not stain the parasite or RBCs. Middle panel shows competitive binding of anti-*Pf*Ap4AH antibody to infected cells, where anti-*Pf*Ap4AH antibodies were incubated with recombinant *Pf*Ap4AH protein in 1:5 ratio. *Pf* D-tyrosyl-tRNA^Tyr^ deacylase (DTD) is a cytoplasmic marker. Lower panel shows non-mitochondrial localization where mtochondria are stained in red. (**C**) RBC membrane localization of *Pf*Ap4AH during trophozoite and schizont stages of parasite. VarC is a marker for infected RBC membrane localization. (**D**) A field view of anti-*Pf*Ap4AH antibody staining of infected RBCs. Significant fraction of cells (~50%) showed membrane localization of *Pf*Ap4AH - here cell is marked with white arrow. Uninfected RBCs (without DAPI and *Pf*DTD staining here) are unstained. White scale bar in confocal figures is of 5 μm.

Full length *Pf*Ap4AH enzyme was expressed in *E. coli* and purified to homogeneity. Gel permeation chromatography results on a calibrated column suggested that the protein is a monomer of ~18 kDa (Fig. 1B). Protein A affinity chromatography purified specific anti-*Pf*Ap4AH antibodies recognised recombinant protein, but did not cross-react with uninfected RBC proteins (Fig. 1C). We also did not observe any signal in our competitive western experiments where purified antibodies were pre-incubated with purified recombinant *Pf*Ap4AH protein in varying molar ratios and used to probe parasite lysate (1:1 ratio data shown) (Fig. 1C). In addition, pre-immune sera failed to detect any protein signal using parasite lysate, suggestive of specific antibody generation against *Pf*Ap4AH (Fig. 1C). However, when the protein was probed in parasite lysate using these antibodies a high migrating band was observed, possibly indicating post-translational modification(s) (Fig. 1C). In order to test the predicted nuclear localization of *Pf*Ap4AH, we performed the confocal microscopy experiments (Fig. 2). We observed that the *Pf*Ap4AH is constitutively expressed during all blood stages of parasites and is non-nuclear (Fig. 2A). Competitive confocal immunofluorescence assays, where antibodies were pre-incubated with *Pf*Ap4AH at varying molar concentrations, failed to produce fluorescence, thus validating the specificity of anti-*Pf*Ap4AH antibodies (1:5 ratio data shown) (Fig. 2B). To assess if *Pf*Ap4AH is mitochondrial (as has been reported in some organisms) we tested localization in presence of mitochondrial marker but failed to observe co-localization (Fig. 2B). In these experiments, D-tyrosyl-tRNA^Tyr^ deacylase (DTD) was used as cytoplasmic marker^27^. During these investigations, we noted that ~50% cells displayed *Pf*Ap4AH localization on the infected RBC membrane (Fig. 2C). This localization was confirmed by using anti-varC antibodies, (varC is cytoplasmic domain of *Pf* erythrocyte membrane protein 1) as markers for RBC membrane (Fig. 2C)^28^. The protein signal was not a result of cross reactivity with an RBC membrane protein as we did not observe signal in uninfected RBCs (Fig. 2D). Interestingly, although conditional, membrane localization has been observed for human Ap4AH in mast cells^12^.

### *Pf*Ap4AH is weakly inhibited by suramin

**Figure 3:**
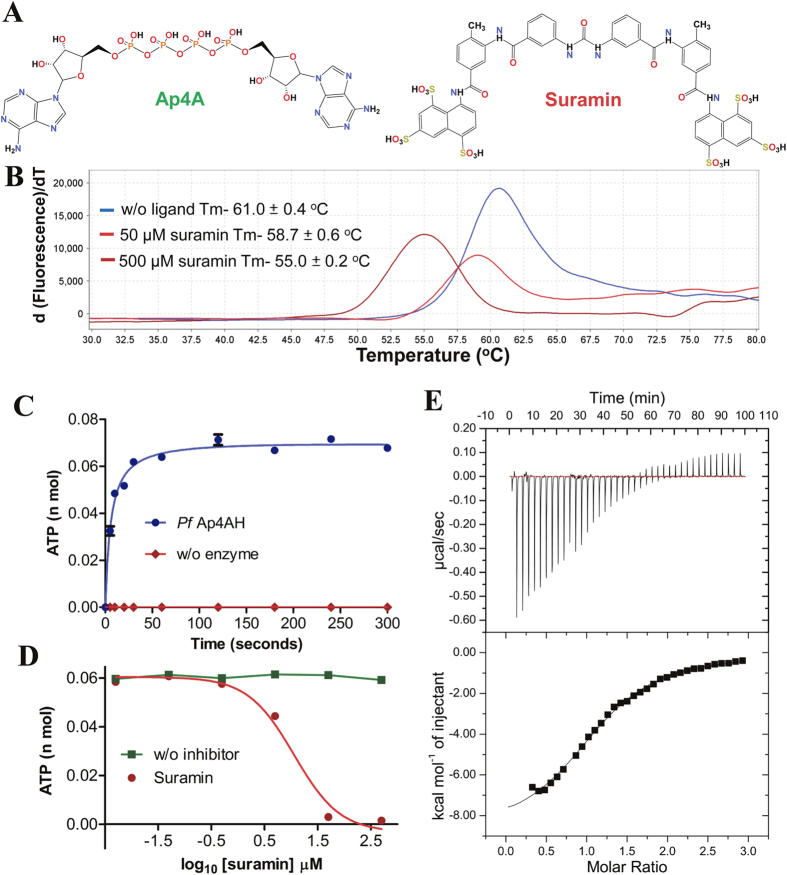
*P. falciparum* Ap4AH is inhibited by suramin. (**A**) 2D structures of Ap4A and suramin are shown. (**B**) Thermal melting profiles of protein without drug (w/o ligand Tm) and in presence of suramin in two molar ratios 1:10 (50 μM suramin Tm) and 1:100 (500 μM suramin Tm) are shown. Negative shift in protein stability was observed in presence of suramin. (**C**) protein enzyme activity curve. Number of ATP molecules produced in nano mole (nano Mol) are plotted on y-axis against different time intervals on x-axis (in seconds). Sample without enzyme (w/o enzyme) was taken as control (**D**) Enzyme activity inhibition in presence of suramin where this drug is used in concentrations ranging from 0.005 to 500 μM for fixed time of 2 minutes. Suramin concentrations are plotted in log scale on X-axis. Enzyme activity without inhibitor is shown in green curve. (*E*) Binding constant for suramin was determined using ITC and Kd value of ~18 μM was obtained. Change in enthalpy caused by suramin titration in buffer alone was subtracted as background from the ligand-binding experiments.

Suramin is a symmetric polysulfonated napthylurea that inhibits *P. falciparum* growth (IC_50_ ~ 10 μM), invasion of RBCs (IC_50_ ~ 60 μM), HepB cells (IC_50_ ~ 50 μM) and was used as remedy for trypanosomiasis and African river blindness (Fig. 3A)^29^. Also, suramin was earlier reported to inhibit rat Ap4AH competitively^30^. We studied the thermal stability profile of *Pf*Ap4AH in the presence of suramin and found that suramin decreased the melting point (Tm) of *Pf*Ap4AH by ~−2.3 °C (50 μM) and ~−6 °C (500 μM) in a concentration-dependent manner (Fig. 3B). The negative shifts indicate suramin binding and stabilization of a partially unfolded *Pf*Ap4AH state^31^. We performed *Pf*Ap4AH enzyme assays to access activity of recombinant enzyme (Fig. 3C), which displayed kinetic parameters similar to the earlier reports (data not shown)^11^. Enzyme assays in the presence of suramin suggested inhibition with an IC_50_ value of ~11.8 μM (Fig. 3D). Isothermal titration calorimetry (ITC) was performed to determine the binding affinity. Favourable hydrogen bonding (ΔH −8817 cal/mol) and hydrophobic interactions (ΔS −7.4 cal/mol) with a binding affinity of ~18 μM and stoichiometry of 1 were observed for suramin and recombinant *Pf*Ap4AH (Fig. 3E) (Table 2).

### Structure determination of *Pf*Ap4AH

**Figure 4:**
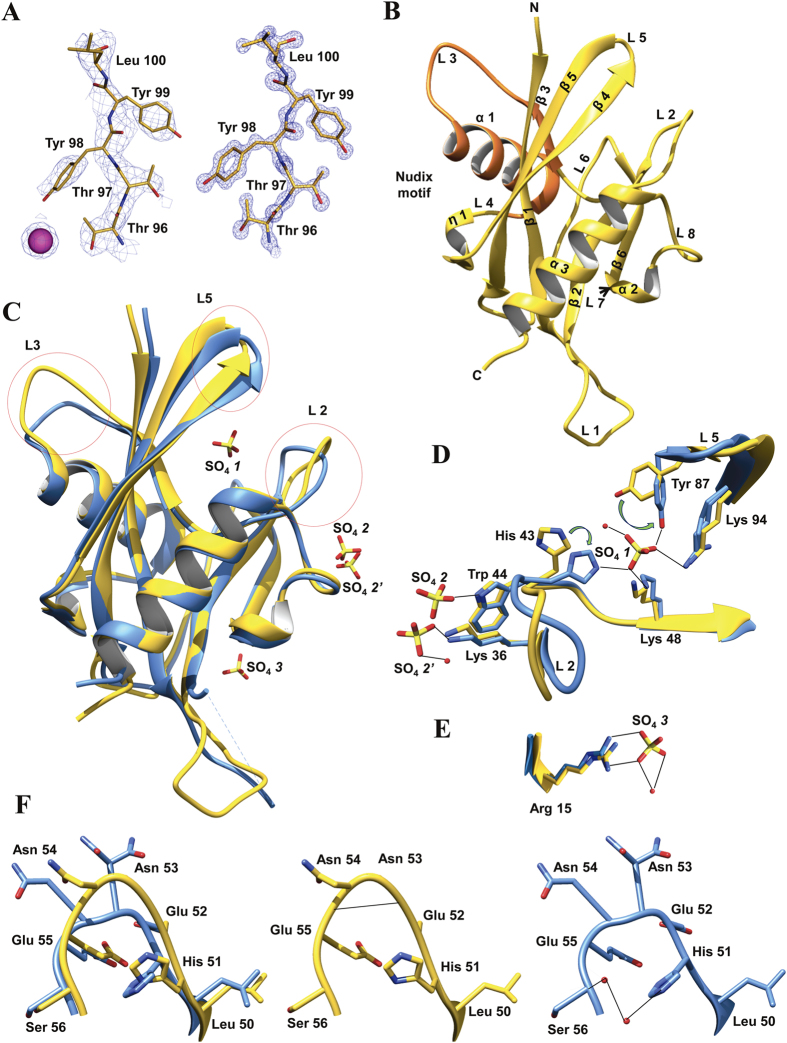
Crystal structure of *Pf*Ap4AH. Residues are denoted by three letter code and adjoining number indicates position in the polypeptide chain. (**A**) First panel shows view of a protein segment showing quality of the model. The experimental electron density is contoured at 5 σ for bound iodide ion (in purple) and at 1.4 σ for protein respectively (3 Å resolution Iodide-SAD data). Second panel is a segment of final model superimposed on 2Fo–2Fc electron density map contoured at 2.5 σ level (**B**) Overall architecture of PfAp4AH structure along with marked secondary structure elements. The Nudix box region(Nudix Motif) is highlighted in orange. (**C**) Superimposition of *Pf*Ap4AH-apo (yellow) and *Pf*Ap4AH-SO_4_ (blue) structures with significant displacement regions circled. Three bound sulphate ions are marked as SO_4_ *1*, SO_4_ *2* (alternative conformer SO_4_ *2’*) and SO_4_ *3*. (**D**) SO_4_ *1* and SO_4_ *2* binding residues and their different rotameric forms are shown. SO_4_ *1* engages the residues (Tyr 87 and Lys 94) present on loop 5 (L5) and His43on loop 2 (L2). Tyr 87 bound to SO_4_ *1* also makes a hydrogen bond with a water molecule. Lys48 is also at hydrogen bonding distance from SO_4_ *1.* SO_4_ *2* and SO_4_ *2’* bind Trp44, water molecule and Lys 36 in *Pf*Ap4AH-SO_4_. The interactions are marked by dotted lines and arrows show direction of sidechain flipping upon SO_4_ binding. (**E**) Interactions of SO_4_ *3* ion. (**F**) Left panel shows superposition of loop L3 in *Pf*Ap4AH-apo (yellow) and *Pf*Ap4AH-SO_*4*_ (blue). Middle and right panels show hydrogen bonding interactions.

Two different crystal structures of *Pf*Ap4AH were obtained by hanging-drop vapour-diffusion method. Our attempts to solve structure using molecular replacement (MR) methods failed, and we used heavy atom soaking method to solve the phase problem. Iodine derivatives were produced by soaking native crystals for 1 min in cryoprotectant solution containing 100 mM NaI. Iodide-SAD data was collected to 3 Å resolution at home source and the anomalous signal was significant only to 4.2 Å resolution. Heavy atom sites were located using SHELXD^32^ and the sites were used for likelihood-based SAD phasing in PHASER for experimental phasing^33^. Initially, 17 iodide sites were located with AutoSol in PHENIX^34^ with a low FOM of 0.34 and these sites were used for phasing. The obtained partial model was fed into AutoBuild for iterative model building and refinement. A total of 534 residues (of the total 608) for 4 molecules in the asymmetric unit were built automatically with R_work_ and R_free_ values of 32 and 39% respectively. The phased map quality is shown in Fig. 4A and relevant statistics are summarized in Table 3. *Pf*Ap4AH apoenzyme (*Pf*Ap4AH-apo from hereon) and suphate bound *Pf*Ap4AH (*Pf*Ap4AH-SO_4_ from hereon) structures were solved using PHASER MR^35^ and one chain of iodide-SAD structure was used as template. Initially, the models were built using AutoBuild in PHENIX. Subsequently, the model was rebuilt manually using COOT^36^ and refined using *phenix.refine* in PHENIX^35^. There are four molecules in asymmetric unit for *Pf*Ap4AH-apo and designated as A, B, C and D. The atomic resolution structure of *Pf*Ap4AH-SO_4_ has three SO_4_ ions and a PEG molecule which arise from crystallization buffer. The quality of the electron density map is shown in Fig. 4A. *Pf*Ap4AH folds into a conventional Nudix domain, with four β-strands (β1, β2, β4 and β5) sandwiched inside two anti-parallel helices (α1 and α3) (Fig. 4B). Overall architecture of *Pf*Ap4AH is similar to the previously reported homologues, such as human (*Hs*Ap4AH; PDB id 3U53)^37^ and *C. elegans* (PDB id 1KT9)^38^ Ap4AHs. The inter-helical angles between two anti-parallel helices (α1 and α3) is 82° and these two helices make an angle of ~38° and ~43° with helix α2 (Fig. 4B). The characteristic Nudix box lies in a region from 48–72 and the active site lies between two loops L2 and L5 (Fig. 4B). Conventionally, polyphosphates in Ap4A molecule are named from P1-P4, where the phosphate attached to a adenine strongly bound Ap4A hydrolase is named as P1^39^. Of the three SO_4_ ions bound in *Pf*Ap4AH-SO_4_, one engages the P1 site (located between loops L2 and L5) (Fig. 4B).

### SO_4_ binding induces conformational changes

Global structural differences between apo- and SO_4_- bound *Pf*Ap4AH were apparent upon superimposition (Fig. 4C). Three major variable regions arising from SO_4_ binding and different space group packing of *Pf*Ap4AH were identified in loop regions L2, L3 and L5 and analyzed further. In the catalytically important loops L2 and L5, a SO_4_ molecule (SO_4_ *1*) was found to bind in the P1 position. (Fig. 4C,D). SO_4_ *1* makes contact with four amino acids in the active site and induces a flip in His43 and Tyr87 side-chains (Fig. 4D). Tyr87 binding to SO_4_ *1* predisposes it to an adenine ring stacking conformation. Other residues involved in hydrogen bonding to SO_4_ *1* are Lys94 (one conformer of the two alternative conformations) and Lys48. His43 binding to SO_4_ *1* leads to changes in loop orientation (L2) of *Pf*Ap4AH-SO_4_ structure. SO_4_ *2* was observed in alternative confirmations, where SO_4_ *2* engages mainly the Trp44 and the alternative conformer SO_4_ *2′* engages Lys36 and a water molecule (Fig. 4D). The SO_4_ *3* is coordinated to a water molecule and a conserved Arg15 (Fig. 4E). Binding positions of SO_4_ *2, 2′* and *3* do not comply with the earlier reported phosphate binding sites elsewhere^37^, and hence may not be relevant for hydrolysis and substrate binding functions of the enzyme. In another major displacement between two structures, the backbone hydrogen bonding keeps the loop L3 in a specific orientation (Fig. 4F). In case of *Pf*Ap4AH-SO_4_, His51 forms a hydrogen bond with one of the water molecule in a nearby water network linked to Ser56 (Fig. 4F). A movie showing overall conformational changes and alterations in interacting residues (within 5 Å distance) of *Pf*Ap4AH upon various ligand bindings is part of supplementary material.

### Sequence alignment and comparison with human structures

**Figure 5:**
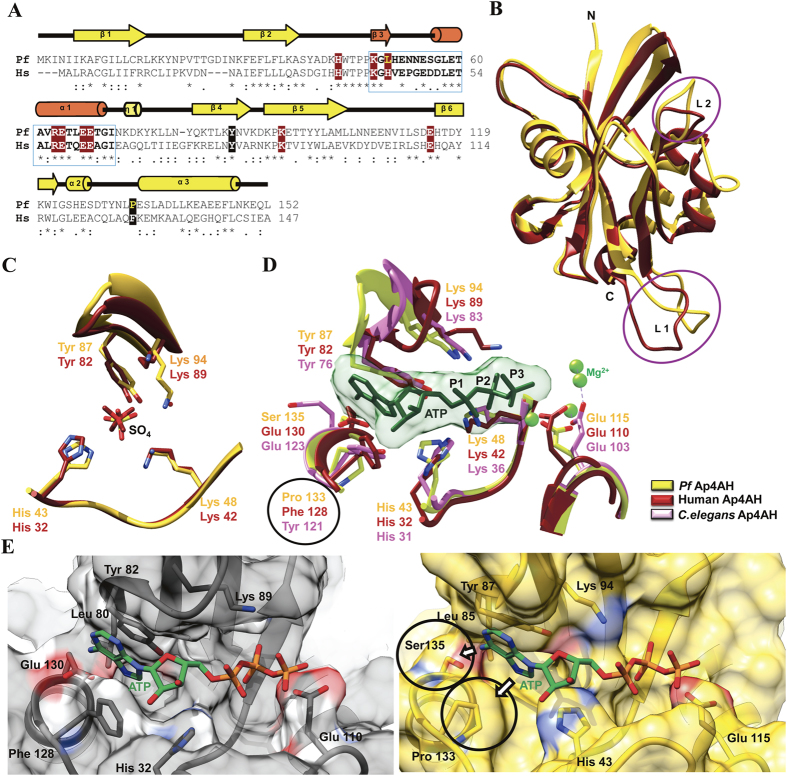
Comparison of human and *P. falciparum* Ap4A hydrolases. (**A**) Structure-based sequence alignment of *P. falciparum* (Pf) and *H. sapiens* (Hs) Ap4AHs with secondary structural elements. Nudix box is highlighted in blue with orange secondary elements. Residues highlighted in red are implicated in phosphate binding while in black are involved in adenine ring accommodation. Key residues in *Pf* differing from human sequence are in yellow, and those marked with asterisk are identical. (**B**) Superimposition of *Pf* (yellow) and Hs (red) Ap4A hydrolases with significantly variable regions circled. (**C**) A view of active site bound SO_4_ ions in Hs (red) and *Pf* (yellow) structures. (**D**) Comparison of ATP (green) binding residues in Hs (red), *C. elegans* (purple) and *Pf* (yellow) structures. Ap4A substrate is accommodated in a negative potential zone of enzyme with help of Mg^2+^ ion, denoted as green spheres. Phosphate groups are marked from P1 to P3, where hydrolysis by the enzyme occurs between P3 and P4 phosphates (not shown) of Ap4A molecule via conserved glutamic acid residue. The ATP molecule is modelled based on superposition of ATP-bound *Hs* structure onto *C. elegans* and *Pf*. Key residue differences are circled. (**E**) Adenine binding pocket of *Pf* and *Hs* Ap4AHs are compared where additional atomic space within *Pf* active site is highlighted.

*Hs*Ap4A hydrolase has sequence identity of ~36% with the *Pf*Ap4AH. Alignment show conservation of key residues implicated in catalysis and binding of substrate (Fig. 5A). Overall 3D architecture of both these proteins is similar with overall root mean square deviation (r.m.s.d.) of 0.88 Å for 110 C^α^-atoms (Fig. 5B). *Pf*Ap4AH contains an insertion of 13 residues in loop region L1 (Fig. 5B) compared to the 10 and 2 amino acid insertions in human and *C. elegans* respectively^37^,^38^. The SO_4_ bound *Pf*Ap4AH-SO_4_ atomic structure is similar to that of sulphate-bound *Hs*Ap4AH structure where a SO_4_ ion is also located in P1 binding site (*Hs*Ap4AH; PDB id 3U53)^37^. We were able to directly compare the active site residues involved in engaging sulphates (or P1 by analogy). Active site-bound SO_4_ is coordinated by analogous residues (*Pf/Hu*) His43/His32, Lys48/Lys42 and Tyr87/Tyr82, but unlike *Pf*Lys94 analogous *Hs*Lys89 does not engage sulphate (Fig. 5C). Structural comparison of *Pf*Ap4AH with known structures of ATP-bound human counterpart^40^ and AMP bound *C. elegans* Ap4AH display a common scheme of substrate engagement and hydrolysis by these enzymes (Fig. 5D). The adenosine ring of substrate is stabilized by π-π stacking interactions with a conserved Tyr on loop 5 and another Tyr/Phe (Fig. 5D). In *Pf*Ap4A hydrolase structure, these two positions are occupied by Tyr87 and Pro133. Ap4A substrate is generally accommodated in a negative charge zone with help of magnesium ions and hydrolysis occurs at 3^rd^ phosphate (P3) by a conserved glutamic acid (Fig. 5D). Presence of Pro133 and Ser 135 in *Pf*Ap4A instead of larger Phe 128 and Glu 130 provides extra space in substrate binding pocket that can be used to design inhibitory compounds that selectively bind *Pf*Ap4AH (Fig. 5E).

## Discussion

The Nudix hydrolase enzyme set present in an organism is often dictated by host metabolic complexity and adaptability^8^. Most intra- and extracellular parasites, including apicomplexans, have either diminished number of hydrolases or none (e.g. mycoplasmas)^8^. Intriguingly, the diverse Nudix enzyme sets in *P. falciparum* and *T. gondii* reported in this study suggest their selective retention post-evolutionary branching (Table 1). Amongst Nudix hydrolases, Ap4AH is a key mediator of invasion and virulence for many bacterial and viral pathogens, especially as Ap4AHs play central roles in bacterial invasion of human RBCs^8^,^17^,^18^. Ap4A and Ap5A molecules, chief substrates of Ap4AH, are key mediators of cellular communication and function through purinergic receptors^8^,^10^,^11^. Hence, signalling mediated by these molecules within RBCs is of special interest in malaria^8^,^10^,^11^ Purinergic signalling has been shown to play role in parasite invasion^41^. Absence of additional domains and presence of *Pf*Ap4AH on infected RBC membrane (Figs 1A and 2) implies that *Pf*Ap4AH has the potential to modulate RBC purinergic signalling and invasion. Intriguingly, we found the *Pf*Ap4AH thermal melting profile to be unusually high (Fig. 3B), a fact that is consistent with the earlier reported high activity of this enzyme at elevated temperatures^11^,^23^,^24^,^25^. It has been reported that erythrocytes, which can synthesize Ap4A on their own, elevate the intracellular levels of Ap4A ~10 fold during heat shock or high temperatures (as occur in blood stage infection of human malaria). Additionally, Ap4A molecule has been shown to regulate haemoglobin functioning^5^,^42^. These observations link with our data that show (a) *Pf*Ap4AH localization on the infected RBC membrane (Fig. 2C,D), and (b) *Pf*Ap4AH’s high thermostability and thermoactvity (Fig. 3B). Hence, it is feasible that *Pf*Ap4AH can access host cell synthesized intracellular as well as extracellular Ap4A and Ap5A molecules, and lower their concentrations - with even higher enzymatic activity during fever conditions (to perhaps tackle higher levels of RBC synthesized Ap4A) and temper Ap4A/Ap5A abundance in the infected RBC. Of further interest is the presence of Ap4A ligase (*Pf*KRS), Ap4AH and HINT1 (PlasmoDB gene id-PF3D7_0817599) within the parasite that suggests possibility of a KRS-Ap4A-Hint1 pathway similar to mammals^5^,^7^. These observations hint at a greater role for *Pf*Ap4AH in parasite biology and our work here establishes a platform for these future investigations. The mechanism of membrane localization for *Pf*Ap4AH (which lacks PEXEL motif) and its post-translation modification (PTM) remains to be determined.

We were able to solve crystal structure of *Pf*Ap4AH in two conformations. These two structures were compared and global changes were mapped for understanding the substrate induced changes (Fig. 4 and supplementary movie). In particular, side chain flip in Tyr87 and His43 suggests substrate-induced conformational adjustment similar to the human counterpart (Fig. 4D and supplementary movie)^37^. Comparative structural analysis of *Hs*Ap4AH and *Pf*Ap4AH shows presence of unoccupied atomic space in *Pf*Ap4AH substrate binding pocket that can be used for designing specific inhibitors to target this enzyme (Fig. 5E). In *Hs*Ap4AH, Phe128 is involved in stacking adenine ring of the substrate, and Glu130 seems to form hydrogen bond with the amino group in adenine of Ap4A. Both these residues are substituted by smaller ones like Pro 133 (for Phe128) and Ser135 (for Glu130) in parasite enzyme at analogous positions. This key difference provides scope for suitable branching in the adenine ring of ATP or Ap4A structural mimics to specifically target the parasite enzyme.

We found that suramin weakly inhibits *Pf*Ap4AH with a K_d_ value of ~18 μM (Fig. 3E). Earlier reports have suggested that suramin targets *Pf* MSP1, *Pf* falcipan-2 and the RBC purinergic signaling pathway, thereby blocking parasite growth invasion and permeability processes in infected RBCs^41^,^43^,^44^,^45^. Here we provide a new link to suramin’s mechanism of action, and propose addition of *Pf*Ap4AH as another suramin target. Taken together, our studies highlight an unexpected localization of *Pf*Ap4AH and its linkage with the RBC purinergic signaling pathway. The structural analyses provide clues to probing this unique enzyme for targeted drug discovery that can subvert the polyphosphate hydrolysis machinery in the parasite.

## Methods

### Identification and annotation of Nudix hydrolases in *P. falciparum* and *T. gondii*

NUDIX hydrolases were probed using HMM-search tool in the HMMR web server (http://hmmer.janelia.org/) by restricting the taxonomy against *T. gondii* and *P. falciparum* and an E-value cut-off of 0.01. Additionally, independent searches for each available Nudix family member annotated in Pfam (id: PF00293) were performed by protein blast in *P. falciparum* (PlasmoDB- http://plasmodb.org/plasmo/) and *T. gondii* (ToxoDB- http://toxodb.org/toxo/) sequence databases. Domains were annotated using SMART^46^, CD –search^47^, superfamily servers^48^ and also by visual inspection of sequence alignments. Localizations were predicted using online servers, MitoProt (mitochondrial localization- http://ihg.gsf.de/ihg/mitoprot.html), WoLF PSORT (nuclear localization- http://wolfpsort.org/) and PATS (for apicoplast localization).

### Cloning, expression, purification and antibody generation

The gene encoding *Pf*Ap4AH (PF3D7_0520600) was cloned into pETM11 vector and expressed in *E. coli* B834 (DE3). For expression, *E. coli* culture was induced at 0.6 OD with 1 mM IPTG and harvested after growth at 18 °C for 20 h post induction. Cells were resuspended in lysis buffer (20 mM Tris pH 8.0, 100 mM NaCl, 5% glycerol, 15 mM imidazole and 2 mM beta-mercaptoethanol (βMe) and lysed by sonication. Supernatant was separated by centrifugation at 16,000 g for 1 h and loaded onto Ni-NTA beads. Protein was eluted using imidazole gradient and purity of fractions was checked on gel. Pure fractions were pooled and His-tag was removed by adding 1 mM DTT, 0.5 mM EDTA and TEV protease (1:50) and incubation for 16 h at 20 °C. Cleaved protein was buffer exchanged overnight to 20 mM Tris (pH 8.0), 40 mM NaCl and 10 mM βMe. Protein was loaded once again to Ni-NTA column to remove uncut protein and TEV protease (which contains non-cleavable N-terminal His tag). Pure protein was collected in flow through. Protein was further purified using gel permeation chromatography (GPC) using a GE HiLoad 10/300 Superdex 75 column in 20 mM Tris pH 8.0, 40 mM NaCl and 10 mM βMe buffer system. Purity was checked once again on SDS PAGE and pure fractions were pooled. Protein was concentrated to 9.5 mg ml^−1^ (A280, extinction coefficient – 24410 M^−1^ cm^−1^) and stored in −80 °C for further use. Pure recombinant protein was provided to Merck (Merck Millipore) for generation of specific protein A affinity chromatography purified anti-*Pf*Ap4AH antibodies in rabbits. These specific antibodies were used for all western and immunofluorescence studies. Recombinant *Pf*Ap4AH (10 ng) was probed in western blot using 1:5000 antibody dilution. Same concentration of pre-immune sera was used in control.

### Confocal microscopy and expression studies

*P. falciparum* 3D7 strain was cultured using human erythrocytes (4% hematocrit) in RPMI-1640 supplemented with 0.5% AlbumaxII (Invitrogen) as previously described^49^. Cells were treated with MitoTracker Red *CMXRos* dye (Invitrogen) for mitochondrial labelling at a final concentration of 50 nM in parasite culture for half an hour. Gametocytes were generated using heparin according the protocol described earlier^50^. Different blood stages of the parasite were fixed and processed for immunofluorescence studies using the protocol described earlier^51^. Briefly, infected RBCs were washed with PBS and fixed using 4% paraformaldehyde and 0.0075% glutaraldehyde in PBS for 30 min at room temperature. After one wash with PBS, fixed cells were permeabilized with 0.1% v/v Triton X-100 in PBS for 10 min. After another PBS wash, cells were treated with 0.1 mg/ml sodium borohydride in PBS for 10 min. Cells were then blocked using 5% w/v BSA in PBS for 1 h and incubated overnight at 4 °C with primary anti-*Pf*Ap4AH antibodies (1:200 dilution). Cells were washed three times for 10 min each with PBS and incubated with AlexaFluor488-tagged or AlexaFluor594-tagged anti-mouse or anti-rabbit secondary antibodies (Invitrogen) for 2 h at room temperature. RBCs were allowed to settle onto Poly-D lysine (50 mg ml^−1^) coated coverslips that were washed three times in PBS, mounted in anti-fade with DAPI (Invitrogen) and then sealed. Nikon A1R microscope with diode (405 nm), argon (488 nm) and helium-neon green (543 nm) was used and 100X oil immersion lens were used in this study. Images were analysed using NIS elements software (version 3.2). Pre-immune serum for each sample was used as control. Anti-*Pf*DTD antibodies, generated in mice, were used as cytoplasmic marker as described earlier^27^ and varC was used as a RBC membrane marker as also described previously^28^. *Pf*Ap4AH recombinant protein was incubated with antibodies (5:1 molar ratio) for 30 min before adding to sample to demonstrate antibody specificity. Infected cells were counted manually under the microscope. To study the native expression, western blot analysis using asynchronous *P. falciparum* (3D7) culture was performed. Infected RBCs were treated with 0.05% saponin to release the parasites followed by washes with PBS till haemoglobin contamination disappeared. Parasite cells were lysed by 3 rounds of freeze-thaw in RIPA buffer (50 mM Tris-HCL, 150 mMNaCl, 1 mM EDTA, 1% NP40, 0.1% SDS, 1% sodium deoxycholate, pH 7.4) containing protease inhibitors cocktail. Parasite lysate was centrifuged and supernatant (25 μg protein) was separated on SDS-PAGE. Proteins were transferred to nitrocellulose membrane and blots were probed using specific anti-Ap4AH primary antibodies (1:1200) and secondary horseradish peroxidase conjugated antibodies (1:1500 dilutions). Bands were visualized using ECL detection kit. Same dilutions of pre-immune sera were used in each case as western controls. Competitive western was performed by incubating purified antibodies with pure protein in molar ratios of 1:1, 1:2 and 1:5 (antibody:protein) prior to western analysis. *P. falciparum* phenylalanine-tRNA synthetase beta subunit (FRS-β) was used as a loading control (probed in 25 μg lysate) using the previously reported protocol^22^. *Pf*Ap4AH was probed in uninfected human RBCs lysate (25 μg) using same procedure and dilutions as for the infected lysate sample.

### Enzyme activity and inhibition assays

*Pf*Ap4AH activity assays and inhibition were performed by detecting ATP (catalysis product) in a luciferase-based bioluminescence assay (ENLITEN ATP Assay kit, Promega) as reported elsewhere^11^. Briefly, a 100 μL reaction volume was used for each reaction in assay buffer 50 mM Tris (pH 7.5), 20 mM NaCl and 5 mM MgCl_2_ with 0.2 nM enzyme at room temperature. Varying substrate concentrations in assay buffer were used to determine kinetics. Ap4A at a concentration of 2 μM was used with varying suramin concentrations (0.005–500 μM, log intervals) to determine IC_50_. 10 μL of reconstituted rL/L reagent (supplied with ENLITEN ATP Assay kit) was added at the end of each reaction and readings were taken on GloMax^TM^ 20/20 luminometer. Moles of ATP produced in each reaction were determined from the ATP calibration curve. Samples without enzyme and substrate were used to subtract background.

### Thermal shift assay

This was performed as reported earlier^52^. *Pf*Ap4AH was diluted in buffer containing 20 mM Tris pH 8.0, 20 mM NaCl and 2x SYPRO orange dye (Life Technologies). Samples containing only protein (5 μM) and protein with suramin (Sigma) at 50 μM and 500 μM were heated from 20° to 96 °C at a rate of 1 °C min^−1^. Fluorescence signals were monitored by StepOnePlus quantitative real-time PCR system (Life Technologies). Each curve was an average of three measurements and was analysed on Thermal shift software (Life technologies) for ΔTm and Tm calculations. Suramin alone in assay buffer was taken as no protein controls and flat line was observed for fluorescence readings at all temperatures. Melt profiles were plotted by instrument software using derivative curve method.

### Isothermal titration calorimetry

ITC experiments were conducted at 30 °C in a MicroCal ITC-200 apparatus (GE Healthcare) and results were analysed using Microcal origin software. *Pf*Ap4AH was prepared in PBS (phosphate-buffered saline) pH 7.4 and suramin was solubilized in PBS buffer. Suramin at a concentration of 1.5 mM was titrated into 100 μM *Pf*Ap4AH. Titrations consisted of a 0.4 μl injection followed by 39 × 1 μl injections with a 120 s interval between injections. Data analyses and peak integration were carried out using Origin 7 software. Titration of suramin in buffer alone was performed to determine the change in enthalpy caused by dilution of the ligand and subtracted as background from actual ligand-binding experiments.

### Crystallization and preparation of iodine derivatives

Crystallization was carried out at 20 °C using hanging drop vapour diffusion method. Crystals were obtained in two conditions: *i*. 1 μl of 0.2 M lithium sulphate, 0.1 M sodium acetate, 3% ethylene glycol, 50% PEG400 and 1 μl of protein (9.5 mg ml^−1^, *Pf*Ap4AH-SO_4_) and *ii.* 1 μl of 20% PEG, 0.3 M potassium nitrate, 0.4 M sodium bromide and 1 μl of protein (9.5 mg ml^−1^, *Pf*Ap4AH-apo). Single plate crystals were added to cryoprotectant (20% glycerol + mother liquor) for one minute before flash freeze in cooled nitrogen gas at 100 K. For phasing crystals were soaked into cryoprotectant solution supplement with 100 mM NaI for 1 min before flash freeze.

### Data collection and processing

Data set for phasing were collected using Cu Kα radiation (λ = 1.54 Å) at 100 K on MAR345 image-plate detector attached on a Rigaku MicroMax-007 rotating-anode X-ray generator operated at 40 kV and 20 mA. A total of 360 images were collected in 1° oscillation steps with 300 s exposure per frame. Diffraction data for crystals of two different conditions (*Pf*Ap4AH-apo and *Pf*Ap4AH-SO_4_) were collected on MARCCD detector at BM14 beam line of European Synchrotron Radiation Facility (ESRF) at Grenoble, France. The diffraction images were processed and scaled with HKL2000 suite programme^53^.

### Phasing, model building and refinement

Iodine SAD data was analysed using SHELXC^54^ and SHELXD^32^ in HKL2MAP^55^. Model was obtained using AutoSol and AutoBuild modules in PHENIX^34^. The atomic (*Pf*Ap4AH-SO_4_) and high (*Pf*Ap4AH-apo) resolution structures were solved using phaser-MR^33^ in PHENIX suit^35^. The models were built manually in COOT^36^ and refined using *phenix.refine*^35^. The quality of all models was checked using PROCHECK^56^ and MolProbity^57^. Structure was analysed and figures were prepared using Chimera^58^ and PyMOL (http://www.pymol.org).

## Additional Information

**How to cite this article**: Sharma, A. *et al.* Structural and functional attributes of malaria parasite diadenosine tetraphosphate hydrolase. *Sci. Rep.* **6**, 19981; doi: 10.1038/srep19981 (2016).

## Supplementary Material

Supplementary Information

Supplementary Movie

## Figures and Tables

**Table 1:** Distribution of putative Nudix hydrolases among *P. falciparum* and *T. gondii*.

Protein	Function	PlasmoDB Gene ID	Localization prediction	ToxoDB Gene ID	Localization prediction
Ap4A Hydrolase	Hydrolysis of Ap4A, Ap5A	PF3D7_0520600	Nucleus	TGME49_214780	Apicoplast, Mitochondria
mRNA decapping enzyme	mRNA decapping	PF3D7_1308900	Nucleus	TGME49_227450	Nucleus
Nucleoside diphosphate hydrolase	Hydrolysis of nucleoside diphosphates linked to other moieties	PF3D7_1349100	Cytoplasm	Absent	–
mRNA cleavage factor-like protein	RNA 3′ processing	PF3D7_0109200	Nucleus	Absent	–
A/G-specific adenine glycosylase, putative	repairing misread A*oxoG residues to C*G by removing the inappropriately paired adenine	PF3D7_1129500	Nucleus	Absent	–
ADP-ribose pyrophosphatase	Hydrolysis of ADP-ribose, ADP-sugar conjugates	Absent	–	TGME49_282190	Cytoplasm
ADP-ribose pyrophosphatase	Hydrolysis of ADP-ribose, ADP-sugar conjugates	Absent	–	TGME49_290900	Mitochondria
Conserved protein	unknown	Absent	–	TGME49_242270	Endoplasmic reticulum

Divergent Nudix hydrolase sets present among apicomplexans *P. falciparum* and *T. gondii* are shown. Proteins were identified using hmmsearch tool in the HMMR web server (http://hmmer.janelia.org/) and by protein blast of Pfam annotated Nudix members (id: PF00293) in *Pf* (PlasmoDB- http://plasmodb.org/plasmo/) and *Tg* (ToxoDB- http://toxodb.org/toxo/) sequence databases.

**Table 2:** Isothermal titration calorimetry data showing binding of suramin to *Pf*Ap4AH.

Temperature °C	K_d_ (μM)	ΔH (cal/mol)	ΔS (cal/mol/deg)	n Value (one site model)
30	18.1 ± 1.1	−8817 ± 192.1	−7.43	1.1 ± 0.0169

**Table 3:** Data collection and refinement settings.

Data set	*Pf*Ap4AH_IOD	*Pf*Ap4AH-apo	*Pf*Ap4A-SO_4_
PDB code	—	5CFI	5CFJ
Data collection		*Pf*Ap4A*-apo*	*Pf*Ap4A*-SO4*
Space group	C121	C121	P2_1_2_1_2_1_
Unit cell dimensions (Å, °)	a = 164.14, b = 64.87, c = 61.46; α = 90, β = 99.53, γ = 90	a = 163.31, b = 64.25, c = 61.41; α = 90, β = 100, γ = 90	a = 31.49, b = 44.34, c = 94.67; α = β = γ = 90
Molecules in ASU	4	4	1
Resolution range (Å)	50.00–3.04 (3.04–2.99)	30.00–2.60 (2.64–2.60)	50.00–1.15 (1.17–1.15)
Unique reflections	13007 (579)	19340 (953)	47576 (2332)
Completeness (%)	99.4	98.3 (97.9)	99.1 (98.6)
I/σ (I)	449/70.3 (70.3/13.6) 6.38 (5.1)	403/29.0 (21.7/14.1) 13.8 (1.5)	684/18.9 (22.5/10.3) 36.2 (2.2)
Rmerge (%)	0.083 (0.346)	0.097 (0.61)	0.040 (0.730)
Redundancy	7.4 (5.5)	5.0 (5.0)	5.3 (5.1)
Corresponding solvent content	47.19%	47.01%	35.54%
Iodine molecules	17	—	—
FOM	0.34	—	*—*
*Refinement*			
B factor	—	54	22
R factor/R free (%)	—	21.0/27.4	17.0/19.3
rmsd in bond lengths (Å)	—	0.009	0.006
rmsd in bond angles (°)	—	1.156	1.144
No. of protein atoms (ASU)	—	4224	1189
No. of water molecules (ASU)	—	68	127
Ligand molecules	—	—	4
*Ramachandran plot*			
Ramachandran favored (*%*)	*—*	95.3	100.0
Ramachandran outliers (*%*)	*—*	0.5	0

^a^Values in parentheses are for the highest resolution shell. ^b^*R*_merge_ = ∑∑|*I*_*hkl*_*-I*_*hkl*_(j)|/∑∑I_*hkl*_, where *I*_*hkl*_*(j)* is the observed intensity and *I*_*hkl*_ is the final average intensity value. ^c^*R*_work_ = ∑∑||*F*_obs_|-|*F*_calc_||/∑|*F*_obs_| and *R*_free_ = ∑||*F*_obs_|−|*F*_calc_||/∑|*F*_obs_|, where all reflections belong to a test set of 5 or 10% randomly selected data. ^d^Root-mean square-deviation from ideal value.
