# Supplementary material for: Structural and functional attributes of malaria parasite diadenosine tetraphosphate hydrolase
Source: Sci Rep. 2016 Feb 1;6:19981. doi: 10.1038/srep19981 (PMC4734340; doi:10.1038/srep19981)
Supplement: Supplementary Information [file srep19981-s1.pdf]

Structural and functional attributes of malaria parasite diadenosine tetraphosphate hydrolase.

Arvind Sharma, Manickam Yogavel and Amit Sharma*

Supplementary video information:

Conformational rearrangement of *Pf*Ap4A hydrolase upon ligand binding. Morph movie showing comparison between apo- and ligand bound- *Pf*Ap4A hydrolase structures is presented. *Pf*Ap4A hydrolase was solved in two conformations. One in Apo- and other bound to three sulfates denoted as SO_4_1, SO_4_2 (alternative conformer SO_4_2'), SO_4_3 and a polyethylene glycol molecule (denoted as PEG). Residues within 5 Å distance of ligand are shown in three letter code.
